# Tolosa-Hunt Syndrome as the Initial Presentation of Systemic Lupus Erythematosus

**DOI:** 10.7759/cureus.61692

**Published:** 2024-06-04

**Authors:** Fathima Nilofar, Kavitha Mohanasundaram, Mahendra Kumar, Gnanadeepan T

**Affiliations:** 1 General Medicine, Saveetha Medical College and Hospital, Chennai, IND; 2 Rheumatology, Prashanth Hospitals, Chennai, IND

**Keywords:** tolosa-hunt syndrome, diplopia, painful ophthalmoplegia, headache, autoimmune disorder, systemic lupus erythematosus

## Abstract

Tolosa-Hunt syndrome (THS), also known as painful ophthalmoplegia, recurrent ophthalmoplegia, or ophthalmoplegia syndrome, is described as severe and unilateral peri-orbital headaches associated with painful and restricted eye movements. THS is an uncommon disorder due to granulomatous inflammation of the cavernous sinus. Although THS is primarily idiopathic, it has rarely been reported in association with systemic lupus erythematosus (SLE). This case report describes a unique case of THS presenting as the initial manifestation of SLE, a multi-system autoimmune disease.

We present a detailed case report of a 54-year-old female patient who presented with THS with the classical symptoms of THS including unilateral headache, double vision, and orbital pain. A cranial nerve examination revealed right oculomotor nerve palsy with the inability to adduct, raise, or depress her right eye. A detailed clinical examination revealed alopecia areata and erythematous macular lesions on her right earlobe. Laboratory investigations were unremarkable except for an increased erythrocyte sedimentation rate (ESR). Diagnostic investigations, including MRI and serological tests, were conducted to explore the underlying causes and systemic involvement.

The patient's MRI showed characteristic findings consistent with THS, while serological tests revealed positive antinuclear antibodies, anti-ds-DNA antibodies, and anti-Smith antibodies and low complement levels leading to a concurrent diagnosis of SLE. There were no other systemic manifestations of lupus at the time of presentation.

Treatment with high-dose corticosteroids led to rapid improvement in ocular symptoms and headaches. Maintenance immunosuppressive therapy was initiated for the management of SLE. The patient had no relapses on follow-up.

This case report underscores THS as a potential initial manifestation of SLE. It highlights the need for comprehensive diagnostic evaluation in patients presenting with atypical cranial neuropathy to consider systemic autoimmune disorders like SLE. Early diagnosis and management are crucial for improving outcomes in such intertwined pathologies. This case emphasizes the need for clinicians to be aware of the possibility of THS as the initial manifestation of SLE.

This extended abstract provides a comprehensive overview of the article, laying out the significance of the case in broadening the clinical understanding of the overlap between localized inflammatory syndromes and systemic autoimmune conditions like SLE.

## Introduction

Tolosa-Hunt syndrome (THS) is a rare inflammatory condition characterized by painful ophthalmoplegia, primarily affecting the cavernous sinus, orbital apex, or both. THS was first described in the year 1954 by Dr. Eduardo Tolosa, a Spanish neurosurgeon [1]. Similar cases were reported by Hunt et al. in 1961 [2]. Smith and Taxdal called it THS for the first time in 1966 [3]. The incidence of THS is one case per million per year. The diagnostic criteria were refined in 2004 by the International Headache Society, such that granuloma, demonstrated by MRI or biopsy, is required for diagnosis [4]. Ophthalmoplegia occurs when the III, IV, and VI cranial nerves are compressed by granulomatous inflammation [5]. Constant orbital pain is caused by inflammation within the cavernous sinus or along the superior orbital fissure. 

Systemic lupus erythematosus (SLE) is a chronic autoimmune disease with diverse manifestations, impacting multiple organ systems with varying degrees of severity. The disease is marked by the production of autoantibodies leading to widespread inflammation and tissue damage. Neuro-ophthalmic involvement in SLE is relatively rare, occurring in about 1-10% of individuals with the disease. It can lead to significant visual impairment and other ocular complications. Neuro-ophthalmic manifestations of SLE may include optic neuritis, cranial nerve palsies, retinal vasculitis, ischemic optic neuropathy, papilledema, and vascular occlusive diseases affecting the eye. Prompt recognition and early management are essential to prevent visual loss and preserve ocular function. THS is one of the rare ophthalmic complications of SLE. 

The occurrence of THS as an initial presentation of SLE is exceedingly rare, while the literature on the subject is limited to isolated case reports. This uncommon presentation challenges clinicians to consider systemic autoimmune diseases in the differential diagnosis of THS, particularly when patients present with atypical features or when THS recurs or persists despite adequate therapy.

This article explores a case of THS that heralded the onset of SLE in a patient without previous lupus diagnosis or typical systemic manifestations, emphasizing the need for high clinical vigilance and interdisciplinary collaboration to unveil underlying systemic conditions such as SLE in patients presenting with isolated THS.

## Case presentation

A 54-year-old female patient sought medical attention due to a one-week history of headaches and a recent onset of double vision and eye pain lasting for two days. She had no history of diabetes mellitus and systemic hypertension. The patient reported no associated symptoms such as vision loss, fever, neck stiffness, trauma, eye redness, or vomiting. Upon clinical examination, she exhibited right oculomotor nerve palsy, characterized by her inability to adduct, elevate, or depress the right eye. The trochlear and abducens nerves remained intact. Other neurological assessments were unremarkable, indicating isolated cranial nerve involvement.

**Figure 1:**
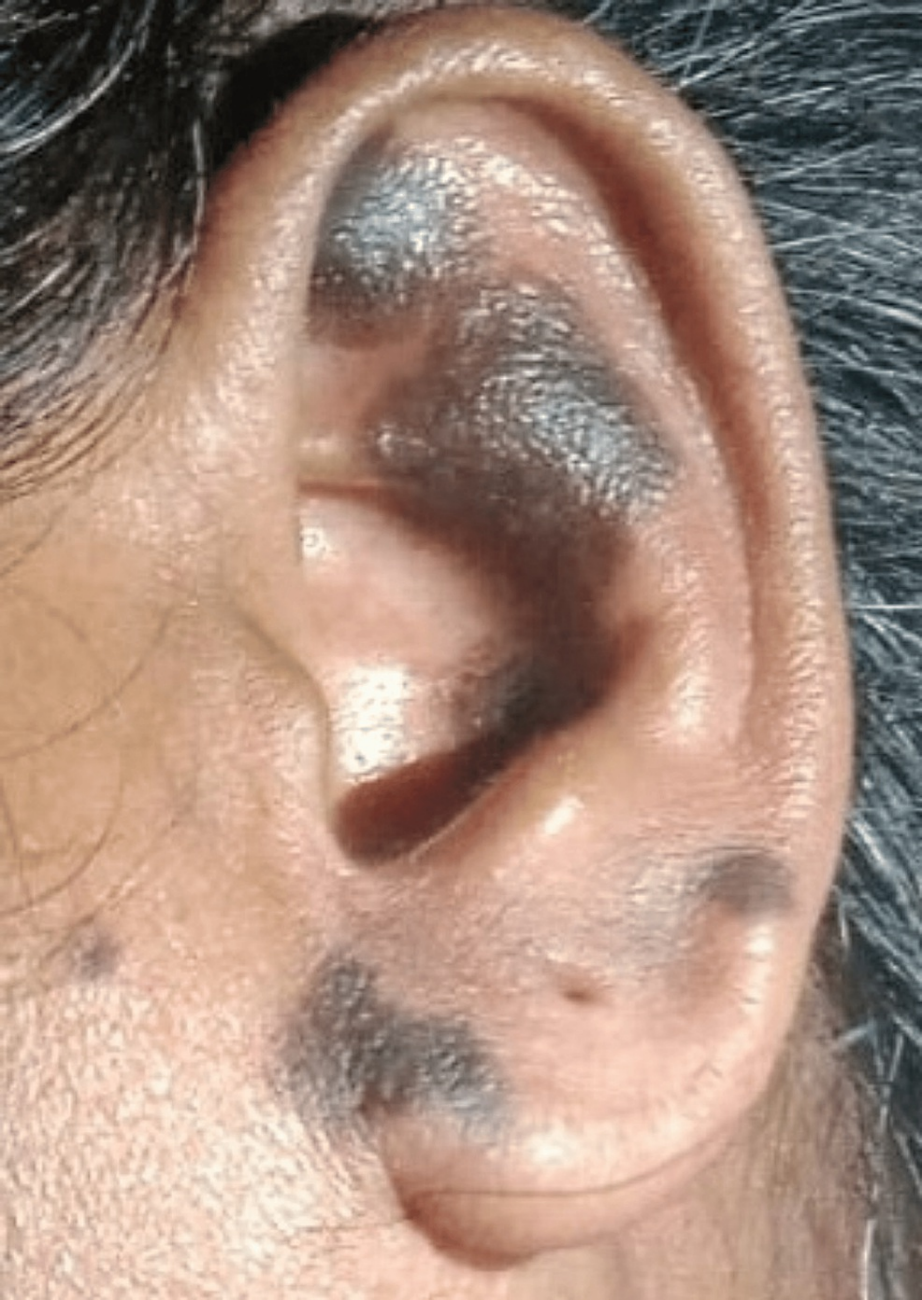
Erythematous macular lesions on the earlobe

**Figure 2:**
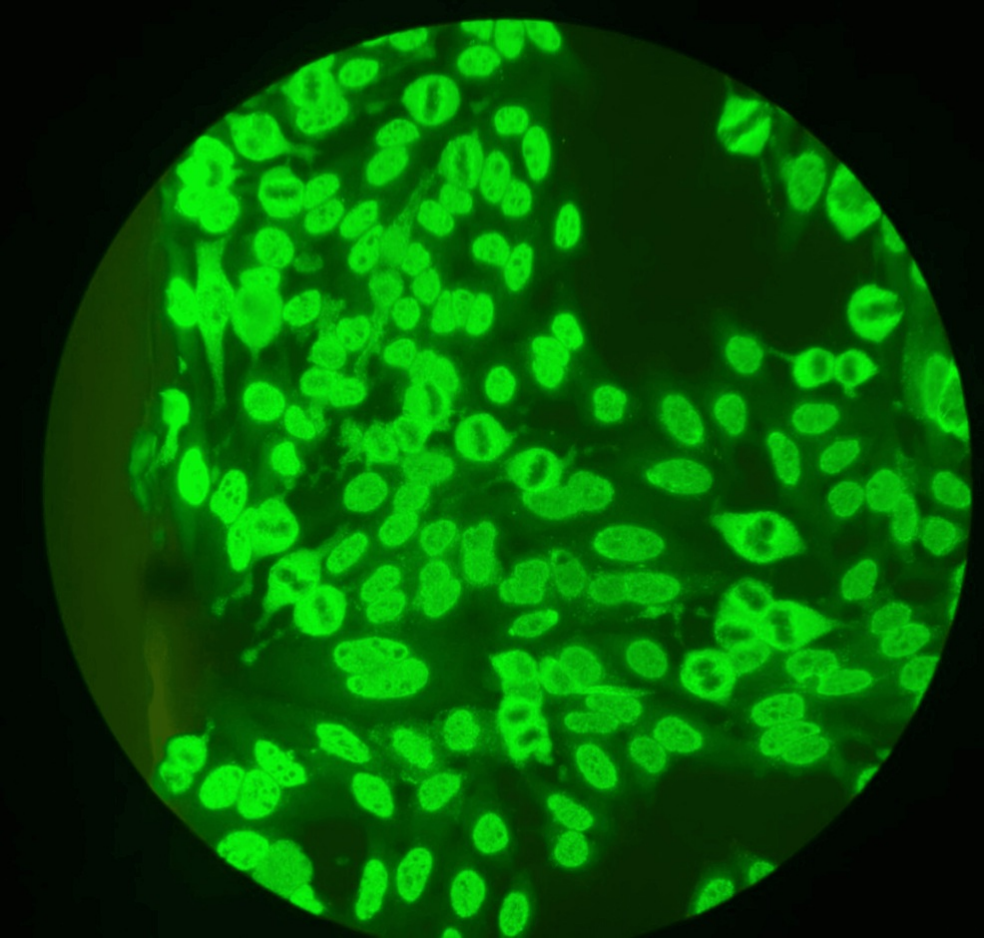
ANA IF done showed a speckled pattern with 4+ positivity ANA IF: antinuclear antibody test by immunofluorescence

The investigations yielded notable findings across various parameters. Hematological analysis revealed an elevated erythrocyte sedimentation rate (ESR) of 84 mm/hr indicating a significant systemic inflammatory response (Table 1). Dermatological evaluation exhibited erythematous macular lesions on the earlobe (Figure 1) and distinct patches of alopecia areata on the back of the scalp, indicative of autoimmune pathology. Serological tests revealed high titres of antinuclear antibodies (ANA) exhibiting a speckled pattern with a 4+ positivity level, indicative of robust autoimmune activity (Figure 2). A cerebrospinal fluid (CSF) analysis was done, and parameters were within normal limits, providing no evidence of central nervous system infection or inflammation (Table 2). ANA immunoblot done showed strong positivity for antibodies suggestive of SLE (Table 3). Neuroimaging through MRI of the brain and orbit showed asymmetric thickening and enhancement of the right cavernous sinus, consistent with THS. These findings, particularly the neuroimaging results and the autoimmune markers, were instrumental in shaping the clinical diagnosis.

**Table 1:** Blood investigations Laboratory parameters revealed elevated ESR suggestive of inflammatory pathology ESR: erythrocyte sedimentation rate; C3: complement component 3; C4: complement component 4; IgG4: immunoglobulin G4; ACE: angiotensin-converting enzyme

Blood investigations	Patient values (normal range)
Hemoglobin	9.4 gm/dl (12-16)
Platelet count	3.33 lakhs/cu.mm (3.5-4.5)
Total leukocyte count	6470/cu.mm (5000-8000)
Absolute lymphocyte count	1018 cells/cu.mm (1000-3000)
ESR	84 mm at 1 hour (less than 20 mm/hour)
C3, C4 level	Normal
Serum IgG4	Normal
Serum ACE level	18 nanomol/mL/min (less than 40)

**Table 2:** CSF analysis CSF analysis done revealed a normal study CSF: cerebrospinal fluid; ADA: adenosine deaminase

CSF analysis	Values (normal values)
Glucose	51 mg/dl (50-80)
Protein	6.78 mg/dl (15-45)
ADA	4.53 IU/L (8.6-28)
Gram smear	No cells, no organisms
Cytology	Acellular smear

**Table 3:** ANA immunoblot test ANA immunoblot test done showed features strongly suggestive of systemic lupus erythematosus ANA: antinuclear antibodies

Antigen	Class
KU	++++
SS-A-2	++
SS-B-3	+++
dsDNA-2	++
Nucleosomes	+++
Histone	++

Based on the clinical presentation of sudden onset double vision, persistent headache, eye pain, and the specific finding of right oculomotor nerve palsy, along with investigative results, a diagnosis of THS was established. The presence of systemic symptoms such as alopecia and skin lesions, along with positive ANA, raised the suspicion of an underlying autoimmune disorder, specifically SLE. This case presents a rare instance where THS is potentially the initial manifestation of SLE, underscoring the complexity of autoimmune diseases and the importance of recognizing unusual clinical presentations in such contexts. The extremely rare incidence of THS and SLE highlights the complexity of autoimmune diseases and the significance of identifying unusual clinical presentations [6]. This particular aspect of the case suggests that autoimmune diseases such as SLE should be taken into account when a patient presents with unusual symptoms of THS. It emphasizes the necessity of a careful and diligent examination when dealing with situations involving the involvement of the cranial nerve and implies that complicated autoimmune diseases might show up in strange ways [7].

The patient's treatment plan is aimed at managing autoimmune symptoms effectively. Pulse steroid therapy was initiated, involving the administration of injectable high-dose methylprednisolone (1 gm daily) for three days to rapidly mitigate acute inflammation and alleviate acute symptoms. Following intravenous steroid therapy, a tapering course of oral steroids was prescribed to maintain inflammation control over a longer period. Hydroxychloroquine was employed as a disease-modifying antirheumatic medication (DMARD), to manage systemic lupus activity [8]. Mycophenolate mofetil was introduced as an immunosuppressive drug to prevent further autoimmune exacerbations, although specific dosing details were not provided [9].

After the initiation of treatment, the patient's symptoms showed improvement in two days and resolved completely in a month, with no THS relapses noted during subsequent follow-up visits. The sustained efficacy of the treatment regimen was evident, and ongoing monitoring was planned to adjust the therapeutic approach as needed based on her clinical response ensuring long-term disease management and stability. This positive outcome highlights the effectiveness of the aggressive and tailored approach in managing complex cases where an autoimmune disorder presents with atypical manifestations such as THS.

This case illustrates the clinical importance of timely and effective treatment of THS and potentially associated autoimmune disorders like SLE, highlighting the need for comprehensive management strategies that address both the immediate- and long-term aspects of such complex diseases.

## Discussion

This case of THS presenting in conjunction with systemic signs suggestive of SLE underscores the complex interplay between localized inflammatory syndromes and systemic autoimmune diseases. The rarity of THS, particularly as an initial manifestation of SLE, provides a unique opportunity to examine the mechanisms that might link these conditions and to reflect on the broader implications for clinical practice.

Based on the International Classification of Headache Disorders (ICHD), the patient's clinical presentation aligns with THS criteria, characterized by unilateral headache, orbital pain, and oculomotor nerve palsy. Diagnostic tests, including neuroimaging and serological markers, further support the diagnosis.

SLE can affect the nervous system with various neurological manifestations, occurring in up to 75% of patients over the course of illness. These effects range from mild cognitive dysfunction and headaches, which are relatively more common, to severe conditions such as neuropsychiatric lupus, occurring in about 30% of cases. Peripheral neuropathy and seizures are reported in approximately 10-20% of individuals with SLE.

THS is typically characterized by granulomatous inflammation of the cavernous sinus, which can cause acute episodes of painful ophthalmoplegia. The underlying aetiology remains poorly understood but is believed to involve autoimmune mechanisms that could be triggered or exacerbated by systemic autoimmune disorders like SLE. In SLE, the immune system produces antibodies that target the body's own tissues, leading to widespread inflammation and damage. The presence of high titres of ANA in this patient, displaying a speckled pattern, is indicative of such autoimmune activity and supports the hypothesis that THS may sometimes be an extra-pulmonary manifestation of SLE.

Diagnosing THS in the context of SLE presents significant challenges due to the overlap of symptoms with other conditions such as infections, malignancies, and other autoimmune diseases. The initial absence of systemic lupus symptoms in this patient highlights the need for a high index of suspicion and comprehensive diagnostic strategies when encountering isolated cranial neuropathies. The integration of detailed imaging studies, comprehensive serological testing, and careful clinical evaluation is crucial for accurate diagnosis and appropriate management.

The management of THS typically involves high-dose corticosteroids to rapidly reduce inflammation, as demonstrated in this case [10]. However, the potential underlying SLE necessitates a more nuanced approach to ensure both immediate symptom relief and long-term management of the autoimmune condition. The use of hydroxychloroquine and mycophenolate mofetil in this patient reflects a strategic approach to modulate the immune response, prevent flare-ups, and manage systemic manifestations [11]. This case highlights the importance of tailored therapy that addresses both the acute and chronic aspects of complex autoimmune presentations.

Further research is needed to better understand the potential connections between THS and systemic autoimmune diseases like SLE. Prospective studies and larger case series provide deeper insights into the prevalence, optimal diagnostic protocols, and most effective treatment strategies for similar cases. Additionally, exploring the genetic, environmental, and immunological factors that contribute to the co-occurrence of these conditions may offer new avenues for targeted therapies and preventive measures.

## Conclusions

This case emphasizes the importance of considering systemic autoimmune diseases in patients presenting with symptoms typical of isolated conditions like THS. It also highlights the critical role of comprehensive, multidisciplinary approaches in diagnosing and managing diseases that manifest across the spectrum of autoimmune and inflammatory disorders. Clinicians must maintain vigilance for atypical presentations of common diseases, as early recognition and intervention are key to improving outcomes in such complex cases. This enhanced discussion delves deeper into the clinical reasoning, the implications of the findings, and the necessary considerations for future research and practice, providing a thorough analysis of the intricate relationship between THS and SLE. This case report reinforces the necessity for clinicians to maintain a broad differential diagnosis when evaluating patients with acute or unusual presentations.
